# Successful bladder-sparing partial cystectomy for muscle-invasive domal urothelial carcinoma with sarcomatoid differentiation: a case report

**DOI:** 10.1177/17562872241226582

**Published:** 2024-01-19

**Authors:** Mark Sultan, Ahmad Abdelaziz, Muhammed A. Hammad, Juan R. Martinez, Shady A. Ibrahim, Mahra Nourbakhsh, Ramy F. Youssef

**Affiliations:** Department of Urology, University of California, Irvine, Orange, CA, USA; Department of Urology, University of California, Irvine, Orange, CA, USA; Department of Urology, University of California, Irvine, Orange, CA, USA; Department of Urology, University of California, Irvine, Orange, CA, USA; Department of Urology, University of California, Irvine, Orange, CA, USA; Department of Pathology, University of California, Irvine, Orange, CA, USA; Department of Urology, University of California, Irvine, 3800 Chapman Avenue, Suite 7200, Orange, CA 92868, USA

**Keywords:** bladder preserving therapy, case report, muscle invasive bladder cancer, partial cystectomy, sarcomatoid urothelial carcinoma

## Abstract

High-grade (HG) urothelial carcinoma (UC) with variant histology has historically been managed conservatively. The presented case details a solitary lesion of muscle-invasive bladder cancer (MIBC) with sarcomatoid variant (SV) histology treated by partial cystectomy (PC) and adjuvant chemotherapy. A 71-year-old male with a 15-pack year smoking history presented after outside transurethral resection of bladder tumor (TURBT). Computerized tomography imaging was negative for pelvic lymphadenopathy, a 2 cm broad-based papillary tumor at the bladder dome was identified on office cystoscopy. Complete staging TURBT noted a final pathology of invasive HG UC with areas of spindle cell differentiation consistent with sarcomatous changes and no evidence of lymphovascular invasion. The patient was inclined toward bladder-preserving options. PC with a 2 cm margin and bilateral pelvic lymphadenectomy was performed. Final pathology revealed HG UC with sarcomatoid differentiation and invasion into the deep muscularis propria, consistent with pathologic T2bN0 disease, a negative margin, and no lymphovascular invasion. Subsequently, the patient pursued four doses of adjuvant doxorubicin though his treatment was complicated by hand-foot syndrome. At 21 months postoperatively, the patient developed a small (<1 cm) papillary lesion near but uninvolved with the left ureteral orifice. Blue light cystoscopy and TURBT revealed noninvasive low-grade Ta UC. To date, the patient has no evidence of HG UC recurrence; 8 years after PC. Patient maintains good bladder function and voiding every 3–4 h with a bladder capacity of around 350 ml. Surgical extirpation with PC followed by adjuvant chemotherapy may represent a durable solution for muscle invasive (pT2) UC with SV histology if tumor size and location are amenable. Due to the sparse nature of sarcomatous features within UC, large multicenter studies are required to further understand the clinical significance and optimal management options for this variant histology.

## Background

Bladder cancer is the tenth most common cancer worldwide.^1^ The largest predictor of bladder cancer mortality is tumor grade with non-muscle-invasive bladder cancer (NMIBC) demonstrating about 95% progression-free survival at 15 years while localized muscle-invasive bladder cancer (MIBC) has a 5-year overall survival of 55%.^2,3^ Neoadjuvant chemotherapy (NAC) followed by radical cystectomy (RC) with pelvic lymph node dissection (PLND) is currently the gold standard for the treatment of localized MIBC. However, these practices are often associated with high morbidity rates, as such we are motivated to durably control early-stage disease while halting progression.

Pure urothelial carcinoma (UC) approximately accounts for 75% of bladder cancer cases, whereas about 25% of cases demonstrate variant histology divided into urothelial and nonurothelial types. Urothelial variants include UC with squamous, glandular, or trophoblastic differentiation, micropapillary, plasmacytoid, tubular and microcystic, nested, clear cell, lymphoepithelioma-like, giant cell, and sarcomatoid types.^4^ Sarcomatoid variant (SV) UC is a rare type of UC of the bladder accounting for 0.1–0.3% of all instances.^5^ By definition, this tumor demonstrates both malignant epithelial and sarcomatoid components. The sarcomatoid component is either spindle cells or can demonstrate heterologous differentiation in the form of rhabdomyosarcomatous, osteosarcomatous, angiosarcomatous, liposarcoma, chondrosarcomatous, or other type of sarcoma.^6^ Due to its rarity and severity, SV UC management presents a substantial challenge. The literature remains limited regarding conservative approaches in the management of these historically aggressive variants.^7,8^ Thus, the American Urological Association (AUA) guidelines recommend early RC in patients with NMIBC with variant histology.^9^

According to the National Comprehensive Cancer Network guidelines, partial cystectomy (PC) is recommended as an option for patients with stage ⩽ cT2 disease and a solitary lesion amenable to segmental resection with adequate negative surgical margins and suitable resulting bladder functional capacity.^10^ The presented case details a solitary lesion of MIBC with sarcomatoid changes treated by PC and PLND with adjuvant chemotherapy without evidence of high-grade (HG) recurrence over 8 years of surveillance. Thus, demonstrating a satisfactory oncological outcome while allowing the patient to maintain bladder function and spontaneous voiding without the need for RC.

## Case presentation

A 71-year-old male with a past 15-pack year smoking history presented to the clinic with a history of MIBC on an outside transurethral resection of bladder tumor (TURBT). His medical comorbidities included hypertension, diabetes, and dyslipidemia; his body mass index was 30.7. Surgical history includes an umbilical hernia repair. The physical exam was noncontributory, and urine cytology returned positive. Imaging by computerized tomography (CT) abdomen and pelvis was negative for pelvic lymphadenopathy or abnormalities in either collecting system. A review of outside pathology was consistent with HG T1 UC but no definite evidence of muscle invasion. Repeat TURBT was recommended, given the need for appropriate staging.

**Figure 1.**
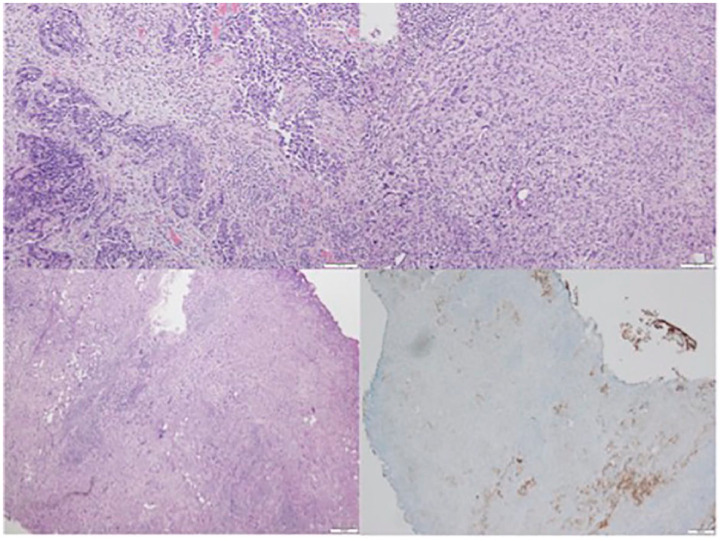
(Left upper) H&E stain (100× magnification) demonstrating invasive HG UC (malignant epithelial component). (Right upper) H&E (100× magnification) demonstrating spindled epithelioid cells with HG pleomorphic nuclei and atypical mitotic figures (resembling pleomorphic HG sarcoma) and consistent with UC. (Lower row) 40× magnification showing sarcomatoid UC with cytokeratin AE1/AE3 antibody (epithelium stain) demonstrating an epithelial to mesenchymal transition (right side). AE1 and AE3, monoclonal antibodies; H&E, hematoxylin and eosin; HG, high grade; UC, urothelial carcinoma.

In the operating room under general anesthesia, complete cystoscopy demonstrated a 2 cm broad-based papillary tumor at the bladder dome, no additional tumors were appreciated. Complete TURBT revealed a final pathology of invasive HG UC with areas of spindle cell differentiation consistent with sarcomatous changes (Figure 1). There was no evidence of lymphovascular invasion and the muscularis propria was present but uninvolved, affirming pT1 disease. For HG T1 urothelial cancer at the bladder dome, the patient was offered different treatment options including RC *versus* PC with or without the need for adjuvant therapy *versus* repeat TURBT with the possibility of intravesical therapies such as Bacillus Calmette–Guerin, if there is no muscle invasion. The patient was inclined toward bladder-sparing options. Given his variant histology portends an aggressive tumor and with shared patient decision-making, an open PC with bilateral PLND was elected.

Under general anesthesia, a midline incision from the umbilicus to the pubic symphysis was accessed to isolate the urachus. Subsequently, bilateral external iliac, internal iliac, and obturator lymphadenectomy was performed without appreciation for any suspicious lymph node morphology. After dissecting the bladder away from the pelvic side wall, direct cystoscopy confirmed the tumor to be confined to the bladder dome. A Satinsky clamp was utilized to isolate the tumor and PC was performed with a 2–3 cm visual margin. A three-layer bladder closure including the mucosa, muscularis, and adventitia was carried out. The abdominal drain was removed on postoperative day 3 while a catheter remained in place for 2 weeks. Final pathology revealed HG UC with sarcomatoid differentiation and invasion into the deep muscularis propria, consistent with pathologic T2bN0 disease with negative margins and no lymphovascular invasion.

The patient subsequently pursued four doses of adjuvant doxorubicin chemotherapy though his treatment was complicated by hand-foot syndrome. Restaging positron emission tomography CT after adjuvant therapy was negative for the disease. In accordance with the AUA guidelines, the patient was screened for recurrence by urine cytology, office cystoscopy, and annual CT with delay phase scans. For the first 2 years after surgery, the patient was screened with urine cytology and office cystoscopy quarterly in addition to an annual CT chest abdomen pelvis with a delay phase. A full timeline of the patient’s management and surveillance is included in Supplemental Table 1. At 21 months postoperatively, the patient developed a small (<1 cm) papillary lesion near but uninvolved with the left ureteral orifice (UO), above the trigone. Given his previous history of HG disease, the patient was counseled for blue light cystoscopy and TURBT.

One hour prior to arriving at the operating room, a catheter was placed to coat the bladder with hexaminolevulinate, which is preferentially absorbed by rapidly dividing cells, allowing for visual identification under UV light. Two lesions were identified, one lesion approximating the left UO as previously described, and another area at the dome of the bladder adjacent to his scar following open PC. After cold cup bladder biopsies, the lesions were resected and fulgurated with bipolar energy. Final pathology revealed noninvasive low-grade Ta UC without muscle involvement of the papillary lesion near the left UO, and reactive epithelium by the bladder dome. The patient continued with screening cystoscopy and urine cytology every 4 months for the first year, then biannually for 2 years with annual upper tract screening by CT after his low-grade recurrence. To date, the patient has no evidence of HG UC recurrence; 8 years after PC. He continues to maintain appropriate bladder function, voiding every 3–4 h with a bladder capacity of about 350 ml.

## Discussion

This case demonstrates the advantage of adequate surgical extirpation (with appropriate margins) in addition to adjuvant chemotherapy as a means for oncologic control of MIBC with SV histological changes. Historically, evidence has supported the SV UC of the bladder (SV-UCB) to be a negative prognostic indicator. A cohort study of 46,515 patients with UC through the Surveillance, Epidemiology, and End Results (SEER) database program in 2007 was reviewed to demonstrate patients with sarcomatoid carcinoma of the bladder presented at a more advanced disease stage as well as have a greater risk for death after adjusting for tumor stage on presentation.^5^ Confirmed again in 2010, the SEER program was analyzed to identify a cohort of 221 patients specifically with SV-UCB, with results demonstrating that SV-UCB presents as HG, an advanced disease with a poor prognosis. The 1-, 5-, and 10-year cancer-specific survival rates were 53.9%, 28.4%, and 25.8%, respectively.^11^ This is a stark contrast to outcomes of RC for MIBC with reported 5- and 10-year cancer-free survival rates at 66% and 68%, respectively.^12^ However, more recent data from 1067 and 624 patient samples with MIBC cancer treated by single tertiary care centers failed to associate the SV with a negative effect on survival after RC.^13,14^ These cohort studies represented data that included 21 and 15 cases of SV-UCB, respectively, between the tertiary care centers, corroborating the paucity of this variant histology. Hence, the current body of data is limited and inconsistent, precluding a full understanding of the disease with relevant randomized controlled trials (RCTs) to establish a gold standard of care.

Previous evidence has recommended forgoing intravesical therapy in patients with SV T1 disease and proceeding directly to RC.^15^ However, the morbidity of RC compared to bladder-preserving approaches may be prohibitive for certain patients, such as the elderly population.^16^ We are therefore motivated to investigate PC as an additional option to manage variant histology UC while preserving adequate bladder and sexual function.^17^ For patients with a solitary lesion amenable to resection, PC allows the surgeon to assess tumor margins completely as well as perform a PLND as needed. Published data from the SEER program registry for stage T1-T2 tumors with variant histology demonstrated no difference in cancer-specific or overall mortality on Cox regression modeling between PC and RC for the treatment of variant histology UC.^18^ In addition, the successful use of PC in conjunction with PLND has previously been reported in the literature to manage SV UC.^19^ Though one limitation regarding PC, as highlighted in this case, is the need for frequent surveillance cystoscopy due to the higher rate of recurrence within native urothelium. Our patient had one recurrence of a small low-grade Ta UCB 21 months after PC.

The recent European Association of Urology guidelines suggest Magnetic Resonance Imaging (MRI) in conjunction with the Vesical Imaging-Reporting and Data System tool to be worthwhile for discriminating between NMIBC and MIBC due to superior soft tissue contrast.^20^ In addition, a pooled meta-analysis of 1724 patients undergoing MRI to stage bladder cancer demonstrated a sensitivity of 0.92 (0.88–0.95) and 0.88 (0.78–0.94) for discriminating between ⩽T1 and ⩾T2 tumors, respectively.^21^ Though MRI was not used in the management of this case, the authors believe clinical T2 disease may likely have precipitated neoadjuvant therapy.

Another consideration is the role of PLND at the time of PC, as performed in this case. A SEER program query published in 2020 on patients with nonmetastatic pT2-T3 UC of the bladder treated by PC discovered only 50% of patients treated by PC concomitantly received PLND. However, the results noted a 5-year case-specific mortality of 30% for patients who received PLND compared to 41% for those who did not (*p* < 0.01).^22^ Though this corroborates the utility of PLND for nonmetastatic MIBC, the data are scarce regarding the utility of PLND for variant histology, particularly SV UC. However, given the patient’s CT imaging for staging demonstrated no evidence of nodal involvement, a standard PLND template was elected over an extended PLND. Intraoperatively, pelvic nodes were not suspected for metastasis.

Endoscopic management as a bladder-preserving option for SV UC has demonstrated inferior overall survival compared to RC.^23^ However, trimodal therapy (TMT) with maximal TURBT, sensitizing chemotherapy, and radiation therapy have been validated as a bladder-preserving solution for MIBC in appropriate patient populations.^24,25^ However, no randomized comparison exists to compare RC to TMT in the management of UC with variant histology. A recent review of 303 patients with MIBC treated by TMT demonstrated the 5-year survival of pure UC at 75%, yet with a small sample size of SV-MIBC cases (*n* = 8), no statistically significant difference was found on subgroup analysis compared to the reported 5-year survival rate of 56% in the SV pathology (*p* = 0.7).^26^

The presented patient was not considered for NAC as the TURBT specimen demonstrated no evidence of muscle invasion and no published data exist to validate a survival benefit with NAC for the treatment of T1 bladder cancer with SV histology, a contrast from evidence for muscle-invasive or metastatic disease. A National Cancer Database study published on patients with non-metastatic MIBC (T2a-T4) demonstrated NAC improved overall survival and pathological downstaging compared to RC alone for patients with SV UC (*n* = 501, *p* = 0.014).^27^ Another SEER registry review of 110 patients with metastatic SV UC reported an overall survival of 8 months with chemotherapy treatment and 2 months without (*p* = 0.016).^28^ Thus, the role of chemotherapy continues to be investigated for the management of SV-UCB.

Current AUA expert opinion guidelines for non-metastatic MIBC with variant histology recommend a divergence from standard evaluation and management.^29^ There is currently no evidence-based consensus for a single appropriate chemotherapy regimen for SV histology. A meta-analysis involving 10 RCTs in patients treated with adjuvant chemotherapy after RC for MIBC (*n* = 1183) demonstrated an absolute 11% improvement in recurrence-free survival (*p* < 0.001) compared to RC alone.^30^ In the presented case, four doses of adjuvant doxorubicin were administered after confirmation of muscle-invasive disease on final pathology. A study by Sui *et al*. compared RC alone (*n* = 106), RC and either chemo- or radiotherapy (*n* = 71), TURBT alone (*n* = 146), and TURBT with chemo- or radiotherapy (*n* = 71) for the treatment of SV UC. Findings validated that patients with RC and some form of multimodal therapy demonstrated the best overall survival.^23^ In addition to the mounting evidence within the literature, this case serves as an anecdotal example to justify the utility of chemotherapy in the treatment of SV-MIBC.

A limitation of this study is the presented utility for PC as a potential management solution for SV pT2 UC is garnered from a single case report. Cohort studies are necessary to delineate oncologic and functional long-term outcomes. Future research endeavors ought to also assess the role of immunotherapy as the sarcomatoid transformation is associated with high PD-L1 expression, and a recent retrospective review of 755 patients with advanced or metastatic disease noted an improved complete response rate for patients with SV UC (52.6%) compared to pure UC (21.1%) after treatment with pembrolizumab (*p* = 0.032).^31,32^

## Conclusion

Surgical extirpation with PC followed by adjuvant chemotherapy may represent a durable solution for muscle-invasive (pT2) UC with SV histology if tumor size and location are amenable for PC. Due to the sparse nature of sarcomatous features within UC, large multicenter studies are required to further understand the clinical significance and optimal management options for this variant histology in the management of bladder cancer. Adequate surgical extirpation with the absence of other aggressive pathological features like lymphovascular invasion and high stage may represent a good prognosis after treatment of UC with SV, as in this case, further promoting cancer-free survival for solitary muscle-invasive UC.

## Supplemental Material

sj-docx-1-tau-10.1177_17562872241226582 – Supplemental material for Successful bladder-sparing partial cystectomy for muscle-invasive domal urothelial carcinoma with sarcomatoid differentiation: a case report Click here for additional data file. Supplemental material, sj-docx-1-tau-10.1177_17562872241226582 for Successful bladder-sparing partial cystectomy for muscle-invasive domal urothelial carcinoma with sarcomatoid differentiation: a case report by Mark Sultan, Ahmad Abdelaziz, Muhammed A. Hammad, Juan R. Martinez, Shady A. Ibrahim, Mahra Nourbakhsh and Ramy F. Youssef in Therapeutic Advances in Urology

sj-docx-2-tau-10.1177_17562872241226582 – Supplemental material for Successful bladder-sparing partial cystectomy for muscle-invasive domal urothelial carcinoma with sarcomatoid differentiation: a case report Click here for additional data file. Supplemental material, sj-docx-2-tau-10.1177_17562872241226582 for Successful bladder-sparing partial cystectomy for muscle-invasive domal urothelial carcinoma with sarcomatoid differentiation: a case report by Mark Sultan, Ahmad Abdelaziz, Muhammed A. Hammad, Juan R. Martinez, Shady A. Ibrahim, Mahra Nourbakhsh and Ramy F. Youssef in Therapeutic Advances in Urology

## Declarations

**Ethics approval and consent to participate:** The need for ethics approval was waived.

**Consent for publication:** Written informed consent for the publication of clinical details and/or clinical images was obtained from the patient. A copy of the consent form is available for review by the Editor of this journal.

**Availability of data and materials:** All clinical data were extracted from the University of California, Irvine Electronic Medical Record System (EPIC). Histopathologic data were acquired from the UCI Department of Pathology. The data are not available to the public under the Health Insurance Portability and Accountability Act (HIPPA). No datasets were generated or analyzed during the current study. The clinical data are however available from the authors upon reasonable request and with permission of the University of California, Irvine.
