# Supplementary material for: Successful bladder-sparing partial cystectomy for muscle-invasive domal urothelial carcinoma with sarcomatoid differentiation: a case report
Source: Ther Adv Urol. 2024 Jan 19;16:17562872241226582. doi: 10.1177/17562872241226582 (PMC10799589; doi:10.1177/17562872241226582)
Supplement: sj-docx-1-tau-10.1177_17562872241226582 – Supplemental material for Successful bladder-sparing partial cystectomy for muscle-invasive domal urothelial carcinoma with sarcomatoid differentiation: a case report [file sj-docx-1-tau-10.1177_17562872241226582.docx]

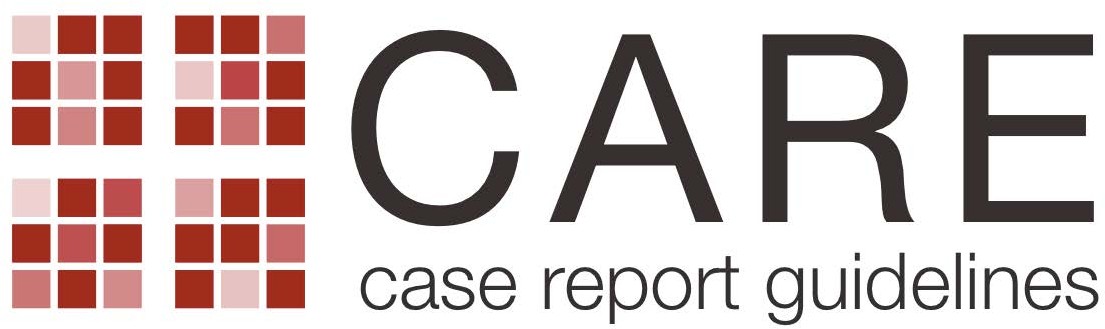
CARE Checklist of information to include when writing a case report
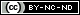


**Topic Item Checklist item description Reported on Line**

**Title 1** The diagnosis or intervention of primary focus followed by the words “case report”

**Key Words 2** 2 to 5 key words that identify diagnoses or interventions in this case report, including "case report"

1-2

46-47

**Abstract**

**(no references)**

**3a** Introduction: What is unique about this case and what does it add to the scientific literature?

**3b** Main symptoms and/or important clinical findings

50-51

54-56

**3c** The main diagnoses, therapeutic interventions, and outcomes

58-60

**3d** Conclusion—What is the main “take-away” lesson(s) from this case?

67-68

**Introduction 4** One or two paragraphs summarizing why this case is unique (**may include** reference**s**)

101-122

**Patient Information 5a** De-identified patient specific information

126-127

**5b** Primary concerns and symptoms of the patient

126-127

**5c** Medical, family, and psycho-social history including relevant genetic information

127-129

**5d** Relevant past interventions with outcomes

126-127

**Clinical Findings**

**Timeline**

**Diagnostic Assessment**

**Therapeutic Intervention**

**Follow-up and Outcomes**

1. Describe significant physical examination (PE) and important clinical findings
2. Historical and current information from this episode of care organized as a timeline

129

Supplementary Table 1

**8a** Diagnostic testing (such as PE, laboratory testing, imaging, surveys).

129-132

**8b** Diagnostic challenges (such as access to testing, financial, or cultural)

**8c** Diagnosis (including other diagnoses considered)

135-138

**8d** Prognosis (such as staging in oncology) where applicable

163-165

**9a** Types of therapeutic intervention (such as pharmacologic, surgical, preventive, self-care)

138-141

**9b** Administration of therapeutic intervention (such as dosage, strength, duration)

156-168

**9c** Changes in therapeutic intervention (with rationale)

142-144

**10a** Clinician and patient-assessed outcomes (if available)

186-189

**10b** Important follow-up diagnostic and other test results

169-176

**10c** Intervention adherence and tolerability (How was this assessed?)

167-169

**10d** Adverse and unanticipated events

174-184

**Discussion 11a** A scientific discussion of the strengths AND limitations associated with this case report

193-210

**11b** Discussion of the relevant medical literature **with references**

212-276

**11c** The scientific rationale for any conclusions (including assessment of possible causes)

288-295

**11d** The primary “take-away” lessons of this case report (without references) in a one paragraph conclusion

288-295

**Patient Perspective 12** The patient should share their perspective in one to two paragraphs on the treatment(s) they received

**Informed Consent 13** Did the patient give informed consent? Please provide if requested . . . . . . . . . . . . . . . . . . . . . . . . . . . . . . . . . . . . . . **Yes No**

X
