# Supplementary material for: Successful bladder-sparing partial cystectomy for muscle-invasive domal urothelial carcinoma with sarcomatoid differentiation: a case report
Source: Ther Adv Urol. 2024 Jan 19;16:17562872241226582. doi: 10.1177/17562872241226582 (PMC10799589; doi:10.1177/17562872241226582)
Supplement: sj-docx-2-tau-10.1177_17562872241226582 – Supplemental material for Successful bladder-sparing partial cystectomy for muscle-invasive domal urothelial carcinoma with sarcomatoid differentiation: a case report [file sj-docx-2-tau-10.1177_17562872241226582.docx]

Supplementary Table 1: Management and Surveillance timeline

| 03/2015 | Outside TURBT, Pathology notable for High Grade Invasive Urothelial Carcinoma |
| --- | --- |
| 05/2015 | Reresection, High Grade Invasive Urothelial Carcinoma with Sarcomatoid Differentiation |
| 07/2015 | Open partial cystectomy with bilateral pelvic lymphadenectomy |
| 11/2015 | Office cystoscopy negative. Cytology negative. Began adjuvant chemotherapy |
| 01/2016 | PET CT negative |
| 03/2016 | Office cystoscopy negative, urine cytology negative |
| 04/2016 | CT Urogram negative |
| 09/2016 | Office cystoscopy negative, urine cytology negative |
| 01/2017 | Office cystoscopy shows edematous/congested region near left UO, urine cytology negative |
| 02/2017 | CT Urogram negative |
| 04/2017 | Office cystoscopy shows small (<1cm) papillary region near the left UO, urine cytology atypical |
| 06/2017 | Blue light cystoscopy and bladder biopsy, pathology returns Low Grade Ta |
| 09/2017 | Office cystoscopy negative, urine cytology negative |
| 01/2018 | Office cystoscopy negative, urine cytology negative |
| 04/2018 | Outside CT CAP negative |
| 05/2018 | Office cystoscopy negative, urine cytology negative |
| 10/2018 | Office cystoscopy negative, urine cytology negative |
| 05/2019 | Office cystoscopy negative, urine cytology negative, CT Urogram negative |
| 02/2020 | Office cystoscopy negative, urine cytology negative, CT Urogram negative |
| 09/2020 | Office cystoscopy negative, urine cytology negative |
| 06/2021 | Office cystoscopy negative, urine cytology negative, renal ultrasound negative |
| 06/2022 | Office cystoscopy negative, urine cytology negative, CT Urogram negative |
